# Environmental Enrichment as a Strategy to Confront Social Isolation Under the COVID-19 Pandemic

**DOI:** 10.3389/fnbeh.2020.564184

**Published:** 2021-01-21

**Authors:** André Davim, Laíse Trindade da silva, Paulo Vieira

**Affiliations:** ^1^Departament of Morphology, Centro Universitário do Rio Grande do Norte, Natal, Brazil; ^2^Febracis Institution, Fortaleza, Brazil

**Keywords:** social isolation, COVID-19, environmental enrichment, neurogenesis, neuroplasticity

## Abstract

The moment of social isolation experienced by the world population due to the COVID-19 pandemic tends to trigger behavioral changes of different orders and on an exponential scale, regardless of social class, age, gender, or ethnicity. Environmental enrichment presents itself as an important strategy to face the social isolation imposed by the pandemic, in order to act as an important agent of induction of biological factors for cognitive and emotional development, favoring a better possibility of adaptation to isolation.

## Introduction

The current moment humanity is going through due to the outbreak of the new coronavirus—COVID-19—imposed an immediate need for confinement to control the epidemiological curve in most countries in the world. According to guidance from health agencies, the World Health Organization (WHO, 2020) being the most representative among them, endorsed and promptly followed up by health entities from all over the world, social isolation of the horizontal type has been showing itself as an effective way to control the progress of COVID-19, with a consequent reduction in morbidity and mortality rates caused by the disease.

Recent studies carried out in several countries find that social isolation presents itself as a catalyst for behavioral changes of the most diverse nature, from anxiety attacks, mild to moderate sadness, and, even in the most extreme cases, episodes of deep depression. Faced with this abrupt and unexpected change in lifestyle, the human kind is put to test with regard to a basic principle of the evolutionary process, which is the phenomenon of adaptation (de Almeida et al., 2008; Grippo et al., 2014).

Studies in human and non-human animals indicate that social stressors, such as social isolation, influence the development of emotional and physiological disorders (Cacioppo and Hawkley, 2003; Grippo et al., 2014). A study of behavior in captive animals, which resembles social isolation in humans, showed that the maintenance of a species in social isolation can be an important tool for the preservation of species, especially in aspects related to biological and behavioral knowledge (de Almeida et al., 2008).

This becomes relevant, as it allows observers to evaluate these aspects. However, the environment in which the animals are inserted can substantially compromise their well-being, and since the environments can be of little sensory stimulus, they are important for the cognitive and emotional development of those individuals. Studies in animal models show that without these stimuli, or yet, being in conditions that do not allow for the expression of specific behaviors, can present significant environment-related behavioral changes, such as excessive irritability, aggressiveness, self-mutilation, hypersexuality, stereotyped movements, apathy, social dullness, and anhedonia, and even develop a depressive state (Mason, 1991; Maestripieri, 1992; Boere, 2001).

## Environmental Enrichment, What is it?

Although we do not have the ability to control the genes we inherit, it is absolutely possible to control the environment around us (Holgate et al., 2017). For this reason, environmental variables have gained prominence, especially when we aim to understand individual strategies for the development and maturation of human beings. There are countless reports in literature discussing the determining role the environment has in promoting the development of healthy habits within most species (Burger and Stice, 2014). Basically, this given enrichment is a combination of two fronts of change in the external environment that occur either individually or in parallel, namely, social and environmental enrichment. Social enrichment concerns interpersonal ties developed with friends, family, and co-workers, which, at the moment, due to isolation, is compromised. Environmental enrichment, on the other hand, concerns the ambient stimuli that are around you, in your place of residence, in your room, at your workplace and/or study grounds.

While social enrichment has the capacity to promote greater ethical and moral reflections on social conflict situations such as poverty, perversity, or human empathy, mediated by ethical and moral issues, environmental enrichment develops the ability to stimulate better behaviors related to the quality of life, such as the development of emotional intelligence, healthier eating, and the practice of regular physical exercises.

Studies on animal models in laboratory, such as amphibians, reptiles, rodents, and some species of non-human primates kept in captivity, are the main sources of experimentation available in the literature on the subject. The term “environmental enrichment” refers to ambient modifications that increase the level of physical stimuli provided by the captive environment, making it more challenging for brain effect.

The objective of enriching the environment is to make the room more complex and interactive, promoting novelties and challenges that simulate situations of a more comprehensive life, thus offering a greater diversity of opportunities for better choice and control of that environment, as well as reducing or preventing the frequency of abnormal behaviors (Boere, 2001). Assessments are unanimous on the positive effects of environmental enrichment on animal health and behavior.

The need for environmental enrichment becomes even more evident when we go through a period of social isolation, such as what we live in this pandemic moment by COVID-19. For example, the study by Holgate et al. (2017) on animals showed that social isolation caused a significant increase in preference for alcohol in animals. However, isolated mice had an even greater preference for alcohol when compared to those who lived alone, but with greater environmental enrichment. Not only for the improvement in behavior related to alcohol consumption, but environmental enrichment can also bring an improvement in engagement for physical activity (Grippo et al., 2014), improvement in food planning, as well as improvement in stress and anxiety levels.

The stimuli of an enriched environment are undoubtedly the external factors that can induce important adaptive behavioral changes, as they induce structural and functional changes at the brain level, quickly and effectively. These stimuli, which improve the environment, are potent agents of induction of biological factors relevant for cognitive and emotional gain, which neurogenesis—formation of new nerve cells—and brain neuroplasticity (Schaeffer, 2010) are part of.

## Neurogenesis and Neuroplasticity

The neurogenesis process, as a concept, refers to the formation of new cells in the central nervous system, with a large number of those formed in the brain from neural and progenitor stem cells (Suzuki et al., 2008). Stem cells are non-specialized cells capable of differentiating into any cells in the body with the ability to self-renew (Zakrzewski et al., 2019).

The nervous system, especially the brain in the adult phase of the individual, presents neural stem cells and neural progenitor cells (Mimeault and Batra, 2006), these cells being directly involved in the process of neurogenesis, creating neurons, astrocytes, and oligodendrocytes, which will play an important role in maintaining physiological integrity, a relevant function for homeostasis of the central nervous system (Hsu et al., 2007; Zakrzewski et al., 2019).

The erroneous idea that new nerve cells are not created after the embryonic development phase came to an end with the discovery of two important areas in the brain of adult mammals where neurogenesis occurs constitutively (Yamashima et al., 2007; Schaeffer, 2010). These areas, the sub-granular zone of the hippocampus and the subventricular zone of the lateral ventricles, are important neurogenic sites with good development potential even at more advanced ages of development. Neurogenesis in the dentate gyrus of the hippocampus in adult individuals is highly regulated by several factors, both environmental and intrinsic to cells to adapt to changes in the environment (Toda et al., 2019).

The discovery that the brain of adult mammals (rodents, monkeys, and humans) can create new nerve cells comes from studies carried out in laboratory animals based on environmental enrichment. Experimental studies show that animals kept in a standardized environment with few sensory stimuli were unable to form new neurons when compared to animals stored in enriched environments. Studies show that the enriched environment, as it presents more complexity and challenges, is able to stimulate the formation of new cells and consequently new neural circuits (Ohline and Abrahan, 2019). This emergence of new neurons becomes extremely important, as it allows animals to change behavioral patterns that, for now, would not be present without proper sources of stimulation.

In terms of brain performance, it is important to understand that with each repetition of a different thought and/or emotion, we reinforce a neural path—and with each new thought, we start to create a new way of being. These small changes, repeated with some regularity in order to maintain constancy, lead to significant changes in how the brain functions. Stimuli such as reading an interesting book, watching a movie, taking a course on an area of interest, participating in a particular game that challenges one's strategic mind, such as a crossword or even a game of chess, or by exposing oneself to different auditory, visual, olfactory, and gustatory stimuli (food) are typical examples of environmental enrichment that stimulate neurogenesis and brain plasticity.

Studies not only show that our brain increases the population of neurons after certain stimuli (Schaeffer, 2010) but also indicate that, if the stimuli do not become habits, therefore repeated over time, around 99% of these new neurons tend to die for lack of stimuli, returning to previous brain performance.

In the postnatal human nervous system, three major neurogenic sites are known—the olfactory bulb and the hippocampus, both located in the brain, and also the cerebellum, although less often discussed (Suzuki et al., 2008). The hippocampus is a brain area that hosts a phenomenon mostly exclusive of the adult mammal brain, which is the addition of new neurons throughout life (Moreno-Jiménez et al., 2019). Once in an enriched environment, sensory stimuli will induce the brain, specifically the hippocampus, to increase its neurogenic activity, which will lead such animals to an important cognitive and behavioral advance. Once the stimuli are more intense and sometimes repetitive, they will naturally lead to the consolidation of memory, thus determining learning.

Our brain is constantly being shaped by the experiences lived as a result of stimuli, and those behavioral changes throughout life occur as a result of brain neuroplasticity, as changes occur in the structure of neurons, such as the appearance of dendritic spikes and collateral axonal branches that allow neurons to increase the capacity of neural connections, thus interfering with the structural and functional organization of the brain as we experience different stimuli, learn new things, and adapt to new changes.

Neuroplasticity can be understood as the brain's ability to modify and adapt, structurally and functionally, in response to the different sensory experiences lived throughout life (Voss et al., 2017). Cerebral neuroplasticity is one of the most enchanting biological events, because from stimuli, whether repeated or under strong emotional impact, structural changes in the neurons that make up that brain may occur, causing neuron dendritic trees, as well as axonal extensions (“arms”) that allow them a greater number of neural synapses with consequent improvement in performance. Let us imagine that a single brain neuron, out of the 86 billion, has the possibility of making up to 10,000 simultaneous synapses. However, this potential will depend exclusively on the quantity and intensity of stimuli for structural changes in neurons to occur, having consequent behavioral changes.

**Figure 1:**
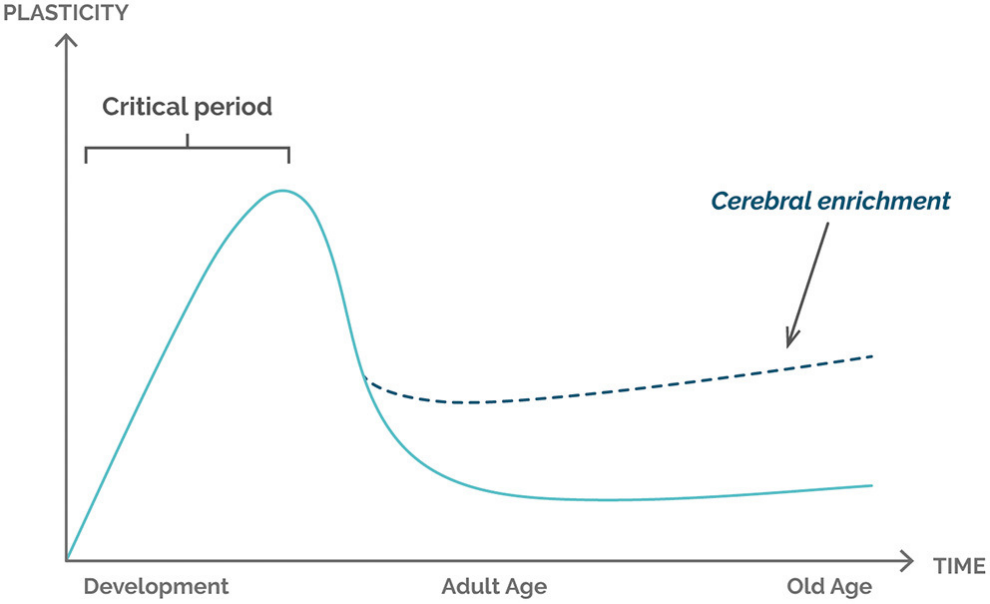
Graphical representation of the relationship between plasticity and life stages of the individual. The critical period represents the phase of greatest brain plasticity, determined by the amount and intensity of new stimuli and greater neurogenesis. Environmental enrichment is shown to be an important factor for increasing plasticity in the adult and senile stages of the individual.

Usually, the brain has a critical period of plasticity that occurs in the early stages of life. The beginning of sensory experiences triggers an opening of a critical time window during which the sensory cortex of the brain is quickly organized and develops in response to passive stimulation from the external environment (enriched environment). This organization refers to cortex alterations including structural changes, since an increase in the cortical layer is observed after stimulations over time. With the development of the individual and the increase in exposure to sensory stimuli, the critical period closes, and the sensory representations tend to stabilize, leading to a decrease in the rate of cerebral neuroplasticity (Figure 1). Cerebral plasticity continues throughout adult life but is now heavily regulated by a variety of cellular and molecular processes, where the enriched environment becomes a primary factor in the later stages of an individual's life.

Regarding the critical period of development of brain plasticity, the study by Johnson et al. (2013) demonstrated the effect of environmental enrichment in rats that suffered brain damage, comparing these results to the practice of physical exercise, notably an effective strategy in the rehabilitation of neural injuries in an animal model. In the study, the animals were kept collectively in an environment enriched with opportunities for various activities. In this environment, the animals had the best results in neuromotor and cognitive tests, demonstrating the effect of environmental enrichment in the rehabilitation process.

## Environmental Enrichment Factors

Studies carried out on captive animals have shown that it is possible that environmental enrichment factors, during periods of social isolation, may attenuate the negative effects of this period from the induced neuroplasticity. For humans, we can mention some factors of environmental enrichment in order to stimulate better brain performance, such as food (Piciotto et al., 2012; Briguglio et al., 2018), regular physical exercise (Rogers et al., 2019), as well as any activity that challenges the brain and is a trigger for the development of neuroplasticity. Studies show that foods rich in precursors and cofactors of neurotransmitters such as acetylcholine, serotonin, dopamine, and γ-aminobutyric acid (GABA) are extremely important for improving brain performance and general performance (Piciotto et al., 2012; Morris, 2016; Haam and Yakel, 2017; Briguglio et al., 2018; Moore et al., 2018). Such neurotransmitters are directly related to relevant behavioral characteristics such as memory, mood (happiness), and also motivation and desire.

It is perceived in relation to serotonin, an important neurotransmitter when it comes to mood and well-being. Tryptophan is the precursor amino acid of this neurotransmitter, being found richly in foods that have as primary source cocoa, such as some chocolates, for example, as well as in almonds, oats, and bananas. Additionally, a good source of tryptophan can be found in kiwi, a fruit of Chinese origin with low glycemic index and widespread in countries with temperate and subtropical climate (Briguglio et al., 2018).

Another important component of the diet that plays a central role in the composition of dopamine is tyrosine, an aromatic amino acid derived from phenylalanine and that can be obtained from the consumption of foods such as some species of fish, nuts, and cheeses (Briguglio et al., 2018). Dopamine acts in an important brain circuit in the brain reward circuit (BRC), which is formed by an evolutionarily old area of the nervous system, the ventral tegmental, and two others, the accumbens nucleus and the prefrontal cortex, more recent. This circuit is dopaminergic and developed over the course of life and is related to motivational behavior and, therefore, important for maintaining the basic activities that lead us to initiate actions.

The gut microbiota is also strongly influenced by environmental factors, factors linked to lifestyle—such as dietary pattern, ingestion of ultra-processed foods, use of antibiotics, and contact with xenobiotics, among others (Levy et al., 2017). These factors can induce the state of dysbiosis, which consists of quantitative or qualitative imbalance in the gut microbiota, causing changes in intestinal function, in the environment, and, consequently, in the microbial behavior of the intestine, which contributes to the manifestation of various diseases (Shanahan, 2012; Louzada et al., 2015; Levy et al., 2017).

Bidirectional communication between the brain and the gut microbiome, known as the microbiota–gut–brain axis, is strongly supported by a vast and growing body of scientific evidence. Studies show that changes in these interactions and their associated behavioral changes imply the pathophysiology of not only classic brain–intestinal communication disorders, such as irritable bowel syndrome and functional gastrointestinal disorders, but also psychiatric and neurological conditions, including mood disorders and affection, anxiety, depression, autism spectrum disorders, neurodegenerative disorders, multiple sclerosis, and chronic pain (Cryan and Dinan, 2012; Fung et al., 2017; Roager and Licht, 2018; Madison and Kiecolt-Glaser, 2019; Osadchiy et al., 2019; Bear et al., 2020).

Such evidence shows the importance and relevance of environmental enrichment manifested in nutritional standards, such as diets rich in fresh foods, fibers, use of probiotics, prebiotics and psychobiotics, omega-3, food poor in processed foods, refined carbohydrates, fat, and chemical additives, in order to modulate and balance the microbial composition of the intestine and, consequently, promote the health of the individual (Wang et al., 2016; Bermúdez-Humarám, 2019; Liao et al., 2019; Bear et al., 2020).

Another factor of environmental enrichment is the regular practice of physical exercise (Rogers et al., 2019), which includes activities in which there is an increase in body stimuli and metabolic rate, stimulating the development of bone and muscle tissues that are essential for weight support, as well as for movements. Regular exercise leads to an improvement in cardiorespiratory activity, which effectively improves the individual's physical performance, since the circulation of oxygen, hormones, and nutrients is better and faster with substances that are fundamental for brain metabolism and cognitive development. Recent studies show that regular physical exercise for, at least, 30 min during the days of the week greatly reduces the risk of cardiovascular disease and improves immunity (Hotting et al., 2016, 2017; Moreno-Collazos and Orti, 2018). An important point to note regarding the practice of physical exercise is that it should not be strenuous, since it was observed in the study by Blain et al. (2019) that excessive exercise (overtraining) causes a reduction in brain activity in the prefrontal lateral cortex, a key region in the cognitive control system, which interferes in decisions, making them more impulsive.

The practice of mindfulness meditation (MM), which is a state of heightened awareness of the body and mind, is yet another factor of environmental enrichment. Numerous studies show that MM contributes and promotes benefits such as the individual's general health and well-being in a sustainable manner, yielding useful insights to guide the treatment of psychological changes/disorders (Tang and Leve, 2016; Larrivee and Echarte, 2018; Berkovich-Ohana et al., 2019).

MM mainly focuses on psychological outcomes, such as behavioral, cognitive, and emotional improvements, and is increasingly incorporated in mental health interventions, proving effective in specific domains of psychopathologies, including depression, anxiety, chronic pain, and substance abuse, as well as emerging efforts related to attention disorders, post-traumatic stress, unregulated eating, and serious mental illnesses (Wielgosz et al., 2019). All of these behavioral changes are a potential target for the emergence or worsening in periods of social isolation such as that experienced by most individuals around the world due to the pandemic imposed by COVID-19.

Studies such as Tang and Leve's (2016) show that countless areas of the brain are activated during the practice of MM, such as those related to the control of attention (anterior cingulate and striatum cortex), those related to the regulation of emotions (prefrontal area, amygdala, and striate cortex), as well as those related to self-awareness and interoception (insula, medial prefrontal cortex, and posterior cingulate cortex).

The insula and the surrounding neural circuits are considered responsible for other functions besides self-awareness, such as the interoception, which is the individual's knowledge about his own physiological and pathological status, for example, with evidence of improvement of this state as a result of neuroplasticity in the insula (Gibson, 2019).

Another viable strategy in terms of EE in situations of social isolation is to create the power posing as a routine habit. The study carried out by researchers from Harvard University and Columbia University showed that when we adopt a power posing through open and expansive postures for a time, important physiological changes occur, such as a decrease in cortisol levels, implying a reduction in the state of stress of the individual, as well as an increase in testosterone levels, promoting an increased feeling of power and risk tolerance (Carney et al., 2010). These findings show that the power posing causes important and adaptive physiological, psychological, and behavioral changes and can be extremely favorable to the individual in times of social isolation and lockdown, becoming a less challenging process and consequently with less negative impacts on the emotions of the population.

## Conclusion

The development of disorders and pathologies, such as alcohol use disorders (AUDs) and severe obesity, involves a complex interaction of genetic and environmental factors (Clarke et al., 2008; Alegria-Torres et al., 2011; Enoch, 2012; Sinha and Jastreboff, 2013), as well as anxiety and depression (Grippo et al., 2014). It is a fact that environmental factors are fundamental to the emergence or suppression of patterns of behavior in individuals in situations of social isolation.

In the specific case of COVID-19, as it is an infectious disease, there was a need for social isolation imposed by health and government entities for the epidemiological control of the disease. From this and due to the prolonged time of this isolation, it is natural that behavioral changes appear as a direct reflection of the isolation, which, many times, alters the routine of people and families living in the same environment. This type of social isolation is different from that where the individual is the active agent in choosing to isolate himself from other people, such as depression. So, the enrichment of the environment becomes an extremely important factor, since the rules that regulate brain plasticity are not only more intrinsically variable as previously thought, as they can be shaped in adult brains, a fact that further enhances the importance of the culture of environmental enrichment in later stages of life, especially in the current COVID-19 pandemic moment and the social isolation imposed as a form of epidemiological control.

Brain plasticity is dependent on sensory experiences lived throughout life and can be enhanced by several factors. Studies such as that of Schaeffer (2010) indicate that non-pharmacological intervention, such as environmental enrichment, may be an appropriate strategy for the promotion of endogenous neurogenesis, thus improving cognitive and behavioral function.

Accumulated evidence suggests that neurons originated in adults may play distinct physiological roles in hippocampal-dependent functions, such as memory coding and mood regulation (Schaeffer, 2010).

Thus, although neuroplasticity is more intense in early stages of development, stimuli from an enriched environment can be adapted and intensified in order to act on plasticity regulators in the adult brain, bringing important cognitive gains, improving performance, and reducing the appearance of mental illnesses such as severe anxiety and depression.

The study by Grippo et al. (2014) showed that environmental enrichment prevented depression and behaviors relevant to anxiety in rats, corroborating the importance of an environment strategy enriched with environmental stimuli as a determining factor for the mental and cognitive health of individuals in social isolation, similar to the experience of the population in the face of the COVID-19 pandemic.

Thus, considering the period of social isolation, one should adopt adaptation measures such as a diet geared to the health of the intestinal microbiota and the brain–intestine axis, consequently favoring a better cognitive and emotional development of the individual, the regular practice of physical exercises with multiple sensory–motor stimuli, the practice of MM, and, depending on the individual's belief, the practice of religious experiences, where studies show that religious and spiritual well-being are important components of not only mental health but also general health (Jakovljevic, 2017; Phillips et al., 2020).

## Data Availability Statement

The raw data supporting the conclusions of this article will be made available by the authors, without undue reservation.

## Author Contributions

AD is the creator of the proposal and wrote most of the article's topics. LT and PV is a co-author and has contributed significantly to the bibliographic review content of the topics covered. All authors contributed to the article and approved the submitted version.

## Conflict of Interest

The authors declare that the research was conducted in the absence of any commercial or financial relationships that could be construed as a potential conflict of interest.
